# Association of serum biomarkers with early neurologic improvement after intravenous thrombolysis in ischemic stroke

**DOI:** 10.1371/journal.pone.0277020

**Published:** 2022-10-31

**Authors:** Yu Cui, Xin-Hong Wang, Yong Zhao, Shao-Yuan Chen, Bao-Ying Sheng, Li-Hua Wang, Hui-Sheng Chen

**Affiliations:** 1 Department of Neurology, General Hospital of Northern Theatre Command, Shenyang, China; 2 Department of Neurology, Haicheng Hospital of Traditional Chinese Medicine, Haicheng, China; 3 Department of Neurology, Chinese People’s Liberation Army 321 Hospital, Baicheng, China; 4 Department of Neurology, Jiamusi University First Affiliated Hospital, Jiamusi, China; 5 Department of Neurology, The Second Affiliated Hospital of Harbin Medical University, Harbin, China; Chinese Academy of Medical Sciences and Peking Union Medical College, CHINA

## Abstract

**Background:**

Early neurologic improvement (ENI) after intravenous thrombolysis is associated with favorable outcome, but associated serum biomarkers were not fully determined. We aimed to investigate the issue based on a prospective cohort.

**Methods:**

In INTRECIS study, five centers were designed to consecutively collect blood sample from enrolled patients. The patients with ENI and without ENI were matched by propensity score matching with a ratio of 1:1. Preset 49 biomarkers were measured through microarray analysis. Enrichment of gene ontology and pathway, and protein-protein interaction network were analyzed in the identified biomarkers.

**Results:**

Of 358 patients, 19 patients with ENI were assigned to ENI group, while 19 matched patients without ENI were assigned to Non ENI group. A total of nine biomarkers were found different between two groups, in which serum levels of chemokine (C-C motif) ligand (CCL)-23, chemokine (C-X-C motif) ligand (CXCL)-12, insulin-like growth factor binding protein (IGFBP)-6, interleukin (IL)-5, lymphatic vessel endothelial hyaluronan receptor (LYVE)-1, plasminogen activator inhibitor (PAI)-1, platelet-derived growth factor (PDGF)-AA, suppression of tumorigenicity (ST)-2, and tumor necrosis factor (TNF)-α were higher in the ENI group, compared with those in the Non ENI group.

**Conclusions:**

We found that serum levels of CCL-23, CXCL-12, IGFBP-6, IL-5, LYVE-1, PAI-1, PDGF-AA, ST-2, and TNF-α at admission were associated with post-thrombolytic ENI in stroke. The role of biomarkers warrants further investigation.

**Trial registration:**

**Clinical Trial Registration**: https://www.clinicaltrials.gov; identifier: NCT02854592.

## Introduction

Stroke is the second leading cause of disability and mortality worldwide, which prevalence increased annually in China [1]. Intravenous alteplase is recommended as the most effective treatment for acute ischemic stroke within 4.5 hours of symptom onset [2]. Early neurologic improvement (ENI) after intravenous thrombolysis is associated with vessel recanalization and predicts functional outcome at 90 days [3,4]. Exploring predictors of ENI is critical for identifying potential patients who can benefit from intravenous thrombolysis.

In the previous studies, female sex, blood glucose < 8 mmol/L, absence of cortical involvement on brain computerized tomography at 24 hours after thrombolysis, leptomeningeal collaterals status, hyperdense artery sign and fall in systolic blood pressure at 24 hours after thrombolysis were found to be associated with ENI in ischemic stroke [5–9]. Given that limited serum biomarkers associated with post-thrombolytic ENI were found [10–13], the issue warrants to be comprehensively investigated.

In INTRECIS (INtravenous Thrombolysis REgistry for Chinese Ischemic Stroke within 4.5 hours of onset) study [14], five centers were selected to consecutively collect blood samples before intravenous alteplase for exploratory analysis. In the exploratory analysis, we measured serum levels of 49 preset biomarkers at admission in thrombolytic patients with ENI versus Non ENI, aiming to detect biomarkers associated with ENI and explore their interactions.

## Methods

### Study population and procedure

From August 2018 to July 2019, patients receiving intravenous thrombolysis within 4.5 hours were consecutively screened to collect blood samples before thrombolysis from five selected hospitals participating in the INTRECIS study. The registry study is nationwide, multicenter, and prospective, which enrolled consecutive adult patients who were eligible for intravenous thrombolysis within 4.5 hours. Details of the trial design and primary outcomes analyses have been previously reported [14]. Briefly, the consecutive patients who received standard dose of alteplase (0.9 mg/kg, maximum 90 mg; manufacturer: Boehringer Ingelheim) within 4.5 hours after symptoms onset were enrolled. The exclusion criteria were as follows: (1) patients received urokinase or non-standard dose of alteplase, (2) patients received any endovascular treatment, (3) lacked complete baseline data, (4) blood samples were not collected before intravenous alteplase. The screened population included in the present study was the same cohort included in our previously published study [15]. However, compared with the previous study, we collected blood samples from different subjects to explore the association between serum levels of biomarkers at admission and post-thrombolytic ENI. According to the occurrence of ENI, patients were divided into two groups: (1) ENI group: patients occurred ENI; (2) Non ENI group: patients did not occur ENI. Furthermore, we performed propensity score matching in groups with the ratio 1:1, caliper of 0.1, nearest-neighbor matching strategy, and operated with control factors including age, gender, smoking, alcohol consumption, medical history, previous use of antiplatelet, blood pressure and glucose at admission, symptom onset-to-treatment time, National Institutes of Health Stroke Scale (NIHSS) score at admission, and the Trial of Org 10172 in Acute Stroke Treatment (TOAST) classification. The trial was centrally approved by the Institution Human Research Ethics Committees of General Hospital of Northern Theater Command with Approval Number k (2016)36-1 and performed in accordance with the Declaration of Helsinki. All the patients and/or their legally gave written informed consent for data collection.

The baseline data of patients were obtained from electronic database: age, gender, smoking, alcohol consumption, medical history, previous use of antiplatelet, blood pressure and glucose at admission, symptom onset-to-thrombolytic time, NIHSS scores, and TOAST classification. Authors had no access to information that could identify individual participants during data collection. ENI was defined as a decrease of ≥4 on the NIHSS scores within 24 hours after thrombolysis from baseline in the present study [2].

### Blood sampling and biomarkers measurements

Before intravenous thrombolysis, four milliliters of peripheral venous blood samples were collected from each patient at admission. The blood samples were centrifuged with the condition of 1000 ⊆ g for 10 minutes at 4°C, and then transferred into 1.8 milliliter cryotube and stored at -80°C until detection.

According to the manufacturer’s instructions, pre-customized protein microarray analysis (Raybiotech Inc) was used to simultaneously detect and quantify serum levels of biomarkers, which were pre-designed based on published data. Identified biomarkers were defined as those variations with p value < 0.05, and fold change > 1.20 or < 0.83. We conducted functional enrichment analysis and protein-protein interactions network to explore mechanism in identified biomarkers

### Statistical analysis

Descriptive statistics was performed to compare variables between groups. Continuous variables with normal distribution were described as means and standard deviation, which included age, blood pressure and glucose at admission, symptom onset to thrombolysis time, NIHSS score, and detected serum concentration of biomarkers. The t tests were used to analyze the normally distributed continuous variables. Categorical variables were described as number and proportions, which included gender, smoking, alcohol consumption, medical history, previous use of antiplatelet, and TOAST classification. The Pearson χ^2^ tests were used to analyze the categorical variables.

The empirical Bayes based linear model method was used to analyze the differential expression of biomarkers between different stages or disease with R package limma. The normalized biomarker values were transformed into log values with base 2, and then input into the analysis. The differential expression was evaluated by adjusted p value (BH method) based on moderated t statistics. In all analysis, differences were considered statistically significant with P value < 0.05. The free statistical language R (version 3.10.3) was used for the outcomes and graph in microarray analysis.

## Results

**Fig 1:**
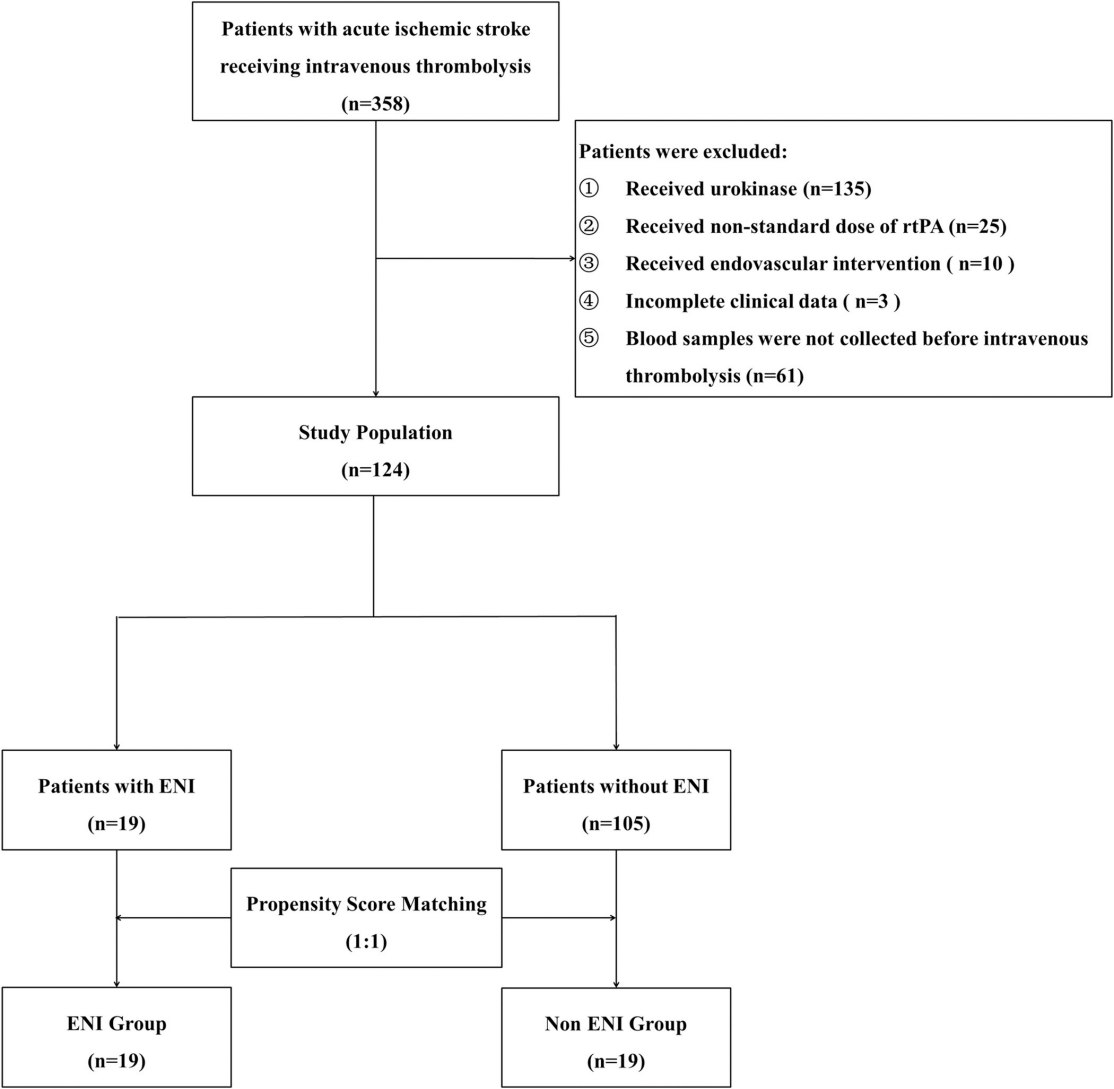
Flow diagram. rtPA, recombinant tissue plasminogen activator; ENI, early neurologic improvement.

Overall, 358 thrombolytic patients were consecutively screened in the current study and 234 patients were excluded due to the following reasons: 135 patients with urokinase and 25 patients with non-standard dose of alteplase, 10 patients with endovascular treatment, 3 patients with incomplete baseline data, and 61 patients without blood samples collection. Finally, 124 patients were included into the study, including 19 patients with ENI, and 105 patients without ENI. With the matching ratio of 1:1, 19 patients without ENI were matched to Non ENI group for analysis (Fig 1). Baseline characteristics showed no significant difference in groups after propensity score matching (Table 1).

**Table 1:** Demographic and baseline characteristics.

Variables	ENI (n = 19)	Non ENI (n = 19)	P Value
**Demographics**			
Age, years, mean ± standard deviation	65.4±9.21	68.4±14.86	0.452
Gender, male, n (%)	7(36.8)	8(42.1)	0.740
Current smoking, n (%)	8(42.1)	11(57.9)	0.330
Alcohol consumption, n (%)	5(26.3)	6(31.6)	0.721
**Medical history, n (%)**			
Stroke	3(15.8)	4(21.1)	0.676
Hypertension	10(52.6)	10(52.6)	1.000
Diabetes mellitus	5(26.3)	5(26.3)	1.000
Atrial fibrillation	4(21.1)	4(21.1)	1.000
Congestive heart failure	3(15.8)	4(21.1)	0.676
Previous use of antiplatelet	2(10.5)	3(15.8)	0.631
**Baseline scales, mean ± standard deviation**			
Systolic blood pressure, mmHg	158.8±24.63	153.0±25.09	0.478
Diastolic blood pressure, mmHg	87.3±9.68	90.0±15.95	0.527
Blood glucose, mmol/L	9.19±4.68	7.68±2.92	0.276
Symptom onset to thrombolysis time, min	160.1±49.11	157.0±53.87	0.854
NIHSS score at admission	9.4±5.69	8.1±5.65	0.462
**TOAST classification, n (%)**			0.185
Large artery atherosclerosis	11(57.9)	10(52.6)	
Small artery occlusion	5(26.3)	4(21.1)	
Cardiogenic embolism	1(5.3)	5(26.3)	
Undetermined cause	2(10.5)	0(0.0)	

ENI, early neurologic improvement; NIHSS, National Institute of Health Stroke Scale; TOAST, the Trial of Org 10172 in Acute Stroke Treatment.

### Biomarkers identification

**Fig 2:**
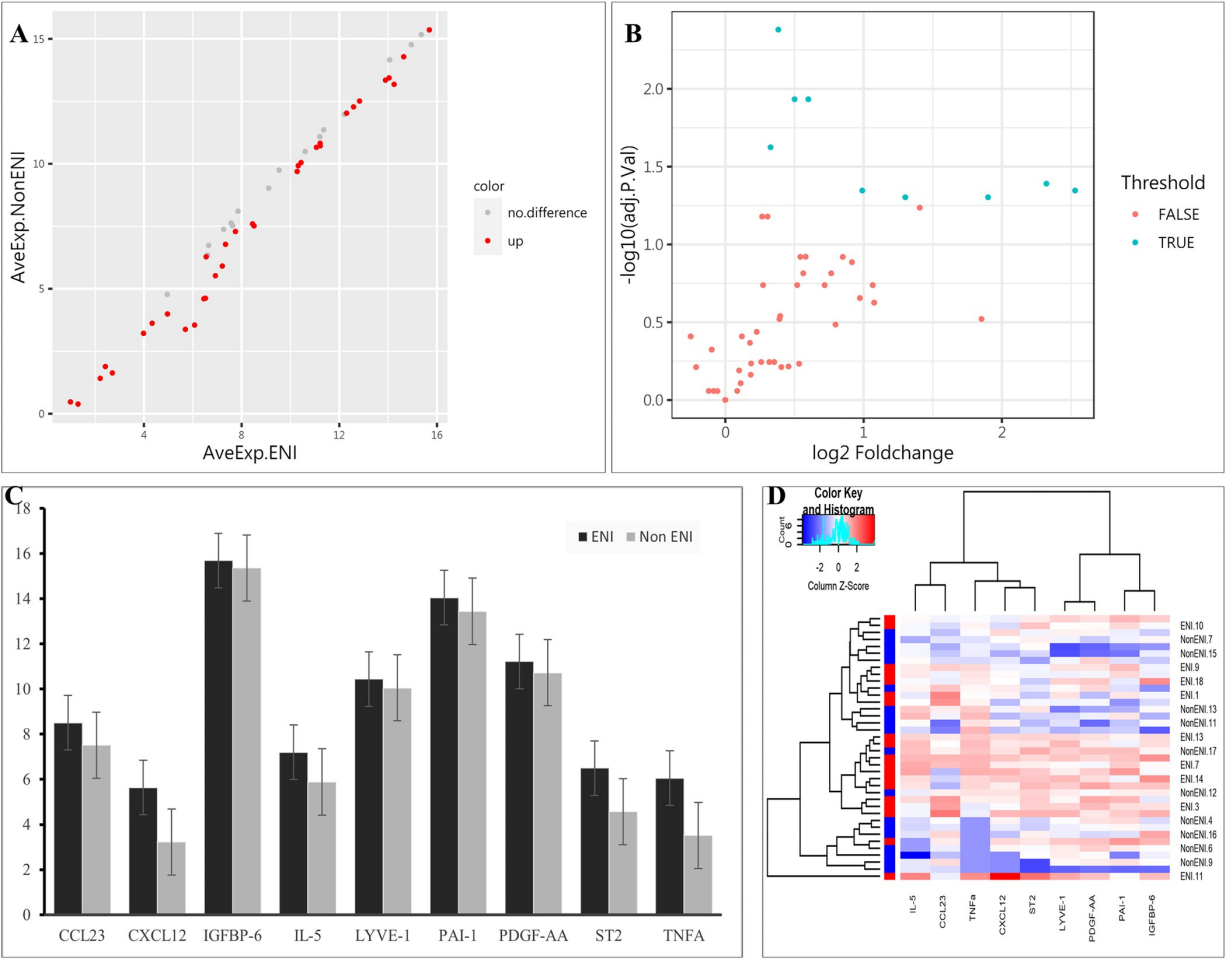
Results of detected biomarkers in the microarray analysis. (A) The scatter plot for detected biomarkers; the X-axis represents the average of log2 serum levels in ENI group, while the Y-axis represents the average of log2 serum levels in Non ENI group; compared with Non ENI group, the gray point represents biomarkers with similar serum levels in ENI group, while the red point represents biomarkers with higher serum levels. ENI, early neurologic improvement. (B) The volcano plot for detected biomarkers; the X-axis represents the log2 foldchange value, while the Y-axis represents the–log10 adjusted P value; the cyan point represents the biomarkers with significant difference, while the red point represents the biomarkers without significant difference. ENI, early neurologic improvement. (C) The column plot for identified biomarkers; the X-axis represents identified biomarkers, the Y-axis represents average of log2 serum levels in groups; the deep color represents ENI group, while the light color represents Non ENI group. CCL-23, chemokine (C-C motif) ligand 23; CXCL-12, chemokine (C-X-C motif) ligand 12; ENI, early neurologic improvement; IGFBP-6, insulin-like growth factor binding protein 6; IL-5, interleukin 5; LYVE-1, lymphatic vessel endothelial hyaluronan receptor 1; PAI-1, plasminogen activator inhibitor 1; PDGF-AA, platelet-derived growth factor AA; ST-2, suppression of tumorigenicity 2; TNFA, tumor necrosis factor α. (D) The heatmap for identified biomarkers; red color represents biomarkers with higher serum levels, while blue color represents the biomarkers with lower serum levels; the darker the color, the more significant the difference of biomarkers. CCL-23, chemokine (C-C motif) ligand 23; CXCL-12, chemokine (C-X-C motif) ligand 12; ENI, early neurologic improvement; IGFBP-6, insulin-like growth factor binding protein 6; IL-5, interleukin 5; LYVE-1, lymphatic vessel endothelial hyaluronan receptor 1; PAI-1, plasminogen activator inhibitor 1; PDGF-AA, platelet-derived growth factor AA; ST-2, suppression of tumorigenicity 2; TNFα, tumor necrosis factor α.

Compared with the Non ENI group, a total of 9 biomarkers with significant difference in ENI group were observed (*P* < 0.05, Table 2). Higher baseline serum levels of chemokine (C-C motif) ligand (CCL)-23, chemokine (C-X-C motif) ligand (CXCL)-12, insulin-like growth factor binding protein (IGFBP)-6, interleukin (IL)-5, lymphatic vessel endothelial hyaluronan receptor (LYVE)-1, plasminogen activator inhibitor (PAI)-1, platelet-derived growth factor (PDGF)-AA suppression of tumorigenicity (ST)-2, and tumor necrosis factor (TNF)-α were identified in ENI group. The results of all the detected biomarkers were shown in the scatter plot and volcano plot (Fig 2A and 2B), while the results of identified biomarker were shown in the heatmap and column plot (Fig 2C and 2D).

**Table 2:** Detected pretreatment serum levels of identified biomarkers.

Biomarkers	Pretreatment serum levels		P Value	Foldchange
	ENI (n = 19)	Non ENI (n = 19)		
**Mean ± standard deviation, pg/ml**				
CCL-23	505.32±416.14	224.57±141.14	0.006	1.99
CXCL-12	1244.39±5178.59	20.33±20.97	0.004	5.00
IGFBP-6	53450.38±8664.92	42904.08±8448.38	0.002	1.25
IL-5	212.50±203.96	96.42±92.19	0.009	2.46
LYVE-1	1389.22±144.92	1089.21±242.02	<0.001	1.30
PAI-1	17523.08±4691.52	11918.16±4343.32	<0.001	1.52
PDGF-AA	2434.21±479.85	1810.86±649.86	<0.001	1.41
ST-2	146.39±198.39	48.56±62.75	0.009	3.73
TNF-α	153.72±221.95	58.29±89.10	0.006	5.77

ENI, early neurologic improvement; CCL-23, chemokine (C-C motif) ligand 23; CXCL-12, chemokine (C-X-C motif) ligand 12; IGFBP-6, insulin-like growth factor binding protein 6; IL-5, interleukin 5; LYVE-1, lymphatic vessel endothelial hyaluronan receptor 1; PAI-1, plasminogen activator inhibitor 1; PDGF-AA, platelet-derived growth factor AA; ST-2, suppression of tumorigenicity 2; TNF-α, tumor necrosis factor α.

### Analysis of function enrichment and protein-protein interaction

**Fig 3:**
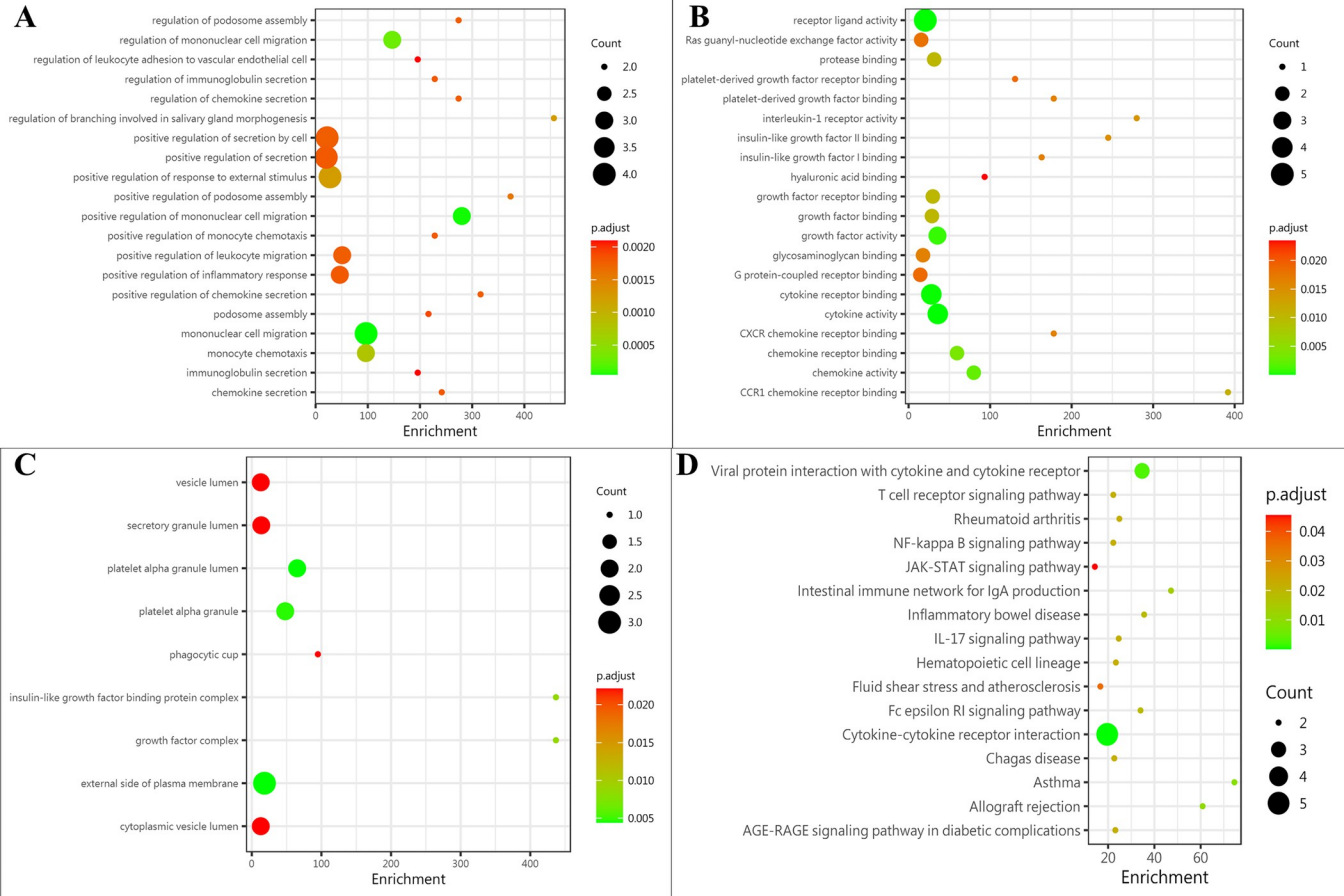
Protein function analysis of identified biomarkers. (A) top 20 significantly enriched biological process of identified biomarkers; the X-axis represents the enrichment, while the Y-axis represents the biological process. (B) the molecular function enriched by identified biomarkers; the X-axis represents the enrichment, while the Y-axis represents the molecular function. (C) the cellular component enriched by identified biomarkers; the X-axis represents the enrichment, while the Y-axis represents the cellular component. (D) the pathway enriched by identified biomarkers; the X-axis represents the enrichment, while the Y-axis represents the pathway. The deeper the color, the larger the P value; the larger the circle, the bigger the counts.

The comprehensive gene ontology (GO) enrichment analysis, which consisted of biological process, molecular function, and cellular component analysis, was conducted to gain a deeper insight into the main functions of the identified biomarkers. The biological process analysis showed that CCL-23, CXCL-12, TNF-α, and PAI-1 were mostly related to the mononuclear cell migration (Fig 3A). The molecular function analysis showed that CCL-23, CXCL-12, TNF-α, IL-5, and PDGF-AA, were mostly associated with the receptor ligand activity (Fig 3B). The cellular component analysis showed that PAI-1 and PDGF-AA, were mostly included in the platelet alpha granule lumen (Fig 3C). The Kyoto Encyclopedia of Genes and Genomes (KEGG) enrichment analysis showed that CCL-23, CXCL-12, TNF-α, IL-5, and ST-2 were included in cytokine-cytokine receptor interaction (Fig 3D).

**Fig 4:**
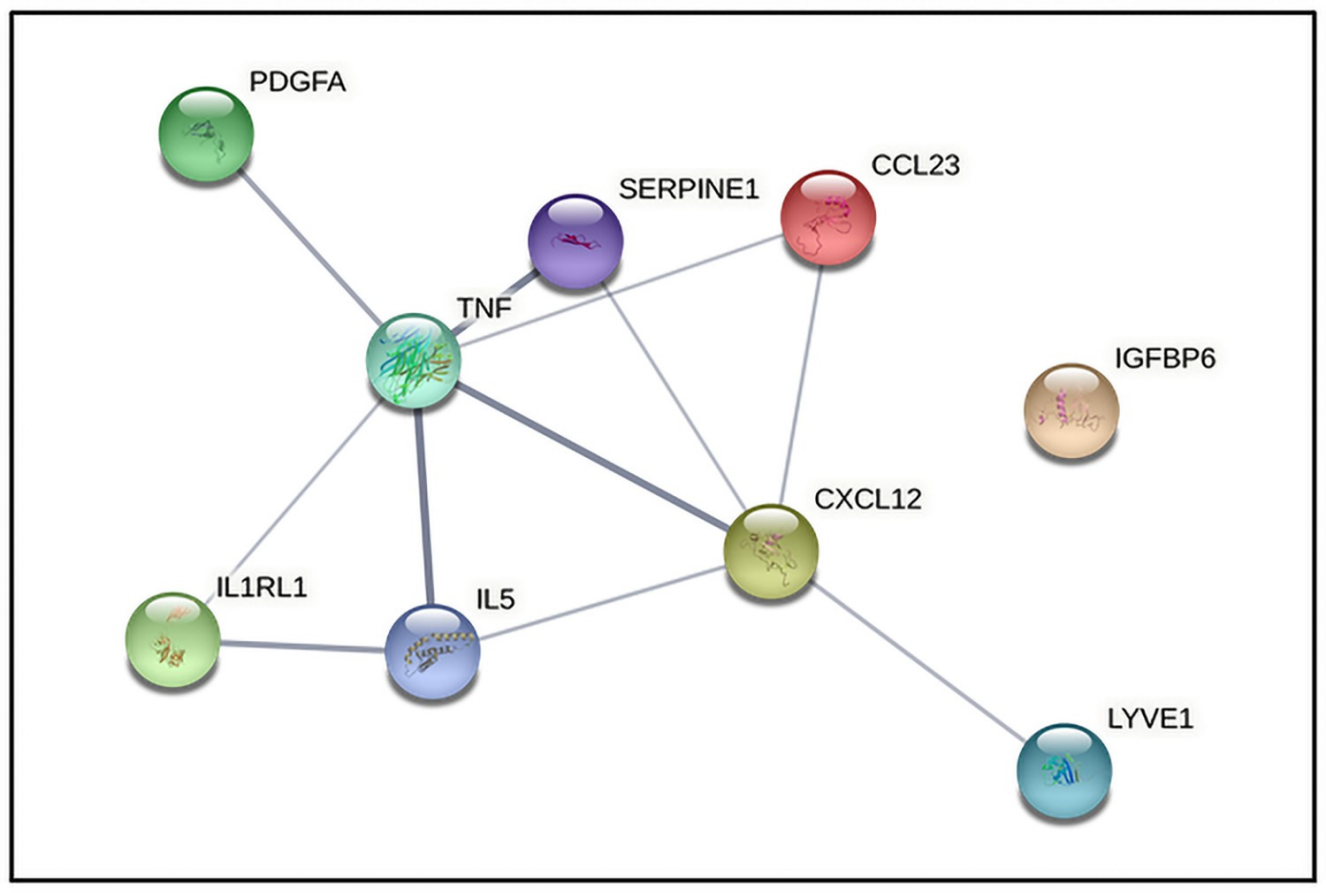
Protein-protein interaction network of identified biomarkers. CCL23, chemokine (C-C motif) ligand 23; CXCL12, chemokine (C-X-C motif) ligand 12; IGFBP6, insulin-like growth factor binding protein 6; IL5, interleukin 5; LYVE1, lymphatic vessel endothelial hyaluronan receptor 1; SERPINE1, plasminogen activator inhibitor 1 (PAI-1); PDGFA, platelet-derived growth factor AA; IL1RL1, suppression of tumorigenicity 2 (ST-2); TNF, tumor necrosis factor α.

According to the information of Search Tool for the Retrieval of Interacting Genes (STRING) database, we build the protein-protein interaction network among identified biomarkers (Fig 4). The results showed that TNF-α (degree = 6) could interact with the most biomarkers, followed by CXCL-12 (degree = 5), IL-5 (degree = 3), CCL-23 (degree = 2), PAI-1 (degree = 2), ST-2 (degree = 2), LYVE-1 (degree = 1) and PDGF-AA (degree = 1).

## Discussion

To our best knowledge, this is the first study to comprehensively investigate serum biomarkers associated with post-thrombolytic ENI by microarray analysis. A total of nine biomarkers were found higher in ENI group than Non ENI group, including CCL-23, CXCL-12, IGFBP-6, IL-5, LYVE-1, PAI-1, PDGF-AA, ST-2, and TNF-α.

Up to date, only a few biomarkers, such as ADAMTS13 activity, Aquaporin-4, leukocyte count, and neutrophil to lymphocyte ratio, were found to be associated with post-thrombolytic ENI in stroke [10–13]. The nine biomarkers identified in the present study were previously reported in ischemic stroke which involved the process of inhibiting development of atherosclerotic plaques, vascular remodeling, neural regeneration, and inflammatory response [16–25]. For example, IGFBP-6 and IL-5 were reported to be associated with inhibiting development of atherosclerotic plaques [26–29], LYVE-1 and PDGF-AA were beneficial to vascular remodeling and neural regeneration, respectively [16–18], while CCL-23, CXCL-12, ST-2, and TNF-α were reported to promote and worsen the inflammatory response: CCL-23, CXCL-12, and TNF-α played pro-inflammatory role [19–21], while ST-2 prevented anti-inflammatory effect of IL-33 [22,23]. In addition, PAI has an effect on inhibiting fibrinolysis in patients treated with tissue plasminogen activator [24,25]. Collectively, these newly identified biomarkers enrich the spectrum of potential serum predictors for ENI in the clinical practice.

Based on functional enrichment analysis, mononuclear cell migration, receptor ligand activity, platelet alpha granule lumen, and cytokine-cytokine receptor interaction were significantly enriched items. After brain injury, CCL-23 and CXCL-12 modulate immune response through promoting migration of monocytes to the local sites of injury [19,20]. As the result of monocytes migration, inflammatory cytokines released from microglia, such as TNF-α, modulate infarct evaluation [21]. It is worthy to note that these cytokines seemed contradictory with ENI given their proinflammatory effects. Taken together, the role of these newly identified biomarkers in ENI is complex and need be further determined given that the elucidation of their complex relationships will provide an insight into our understanding of the mechanisms underlying ENI.

The main strength of this study is the first report of nine biomarkers associated with post-thrombolytic ENI through comprehensively screening preset biomarkers in a prospective cohort. The findings may contribute to further understanding of potential mechanisms underlying early post-thrombolytic outcome improvement in ischemic stroke. However, we recognize several limitations of our study. First, smaller sample size in the present study weaken the power of conclusion. Second, the sensitivity of identified biomarkers for predicting ENI need to be validated in other cohorts with more patients. Third, we could not rule out the effect of an acute phase reaction or comorbidity on these biomarkers. Fourth, in the present study, we only collected the blood sample before thrombolysis and excluded the patients who received mechanical thrombectomy. Therefore, we did not investigate dynamic changes of these biomarkers over the course of follow-up, as well as the effect of reperfusion treatment such as thrombolysis and mechanical thrombectomy on these biomarkers. Last, complex roles of identified biomarkers need further be investigated.

## Conclusion

For the first time, we found that higher serum levels of CCL-23, CXCL-12, IGFBP-6, IL-5, LYVE-1, PAI-1, PDGF-AA, ST-2, and TNF-α at admission were associated with post-thrombolytic ENI in ischemic stroke. The value of these newly identified biomarkers warrants further investigation.
